# Supplementary material for: Coordinating Effect of VEGFC and Oleic Acid Participates to Tumor Lymphangiogenesis
Source: Cancers (Basel). 2021 Jun 8;13(12):2851. doi: 10.3390/cancers13122851 (PMC8227717; doi:10.3390/cancers13122851)
Supplement: Supplementary file 1 [file cancers-13-02851-s001.zip › cancers-1194417-supplementary.pdf]

Supplementary Figure 1

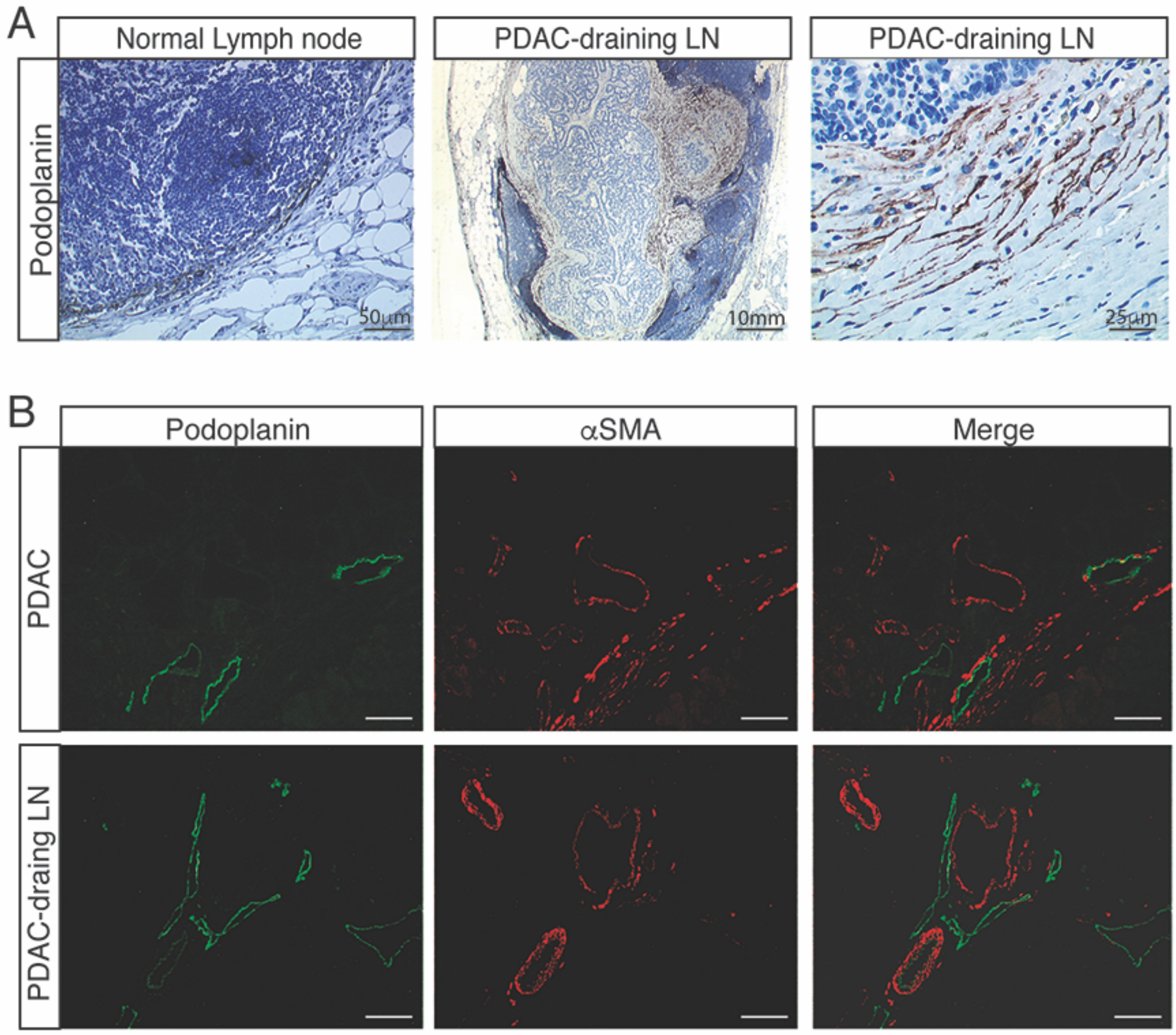

Supplementary Figure 1. Lymphatic vessels invade tumor-draining lymph nodes.
A, Representative images of podoplanin labelling in human PDAC sentinel lymph node (n=15). B, Representative images of podoplanin and alpha-SMA labelling in human PDAC sentinel lymph node(n=15)(Bar=50mm).

Supplementary Figure 2

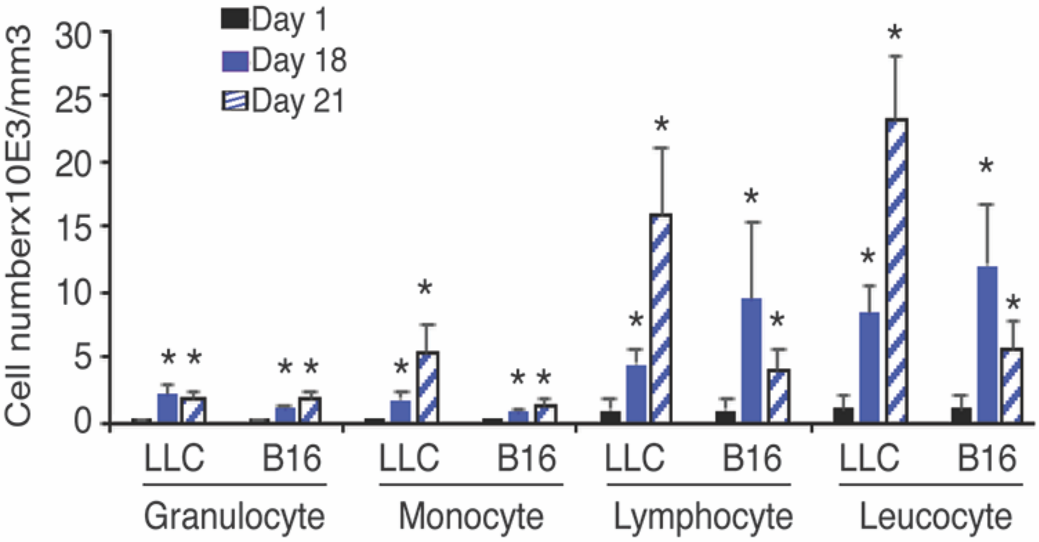

Supplementary Figure 2: Immune cell numeration

Quantification of blood circulating granulocytes, monocytes, lymphocytes and leucocytes in LLC- and B16-tumor bearing mice.

Supplementary Figure 3

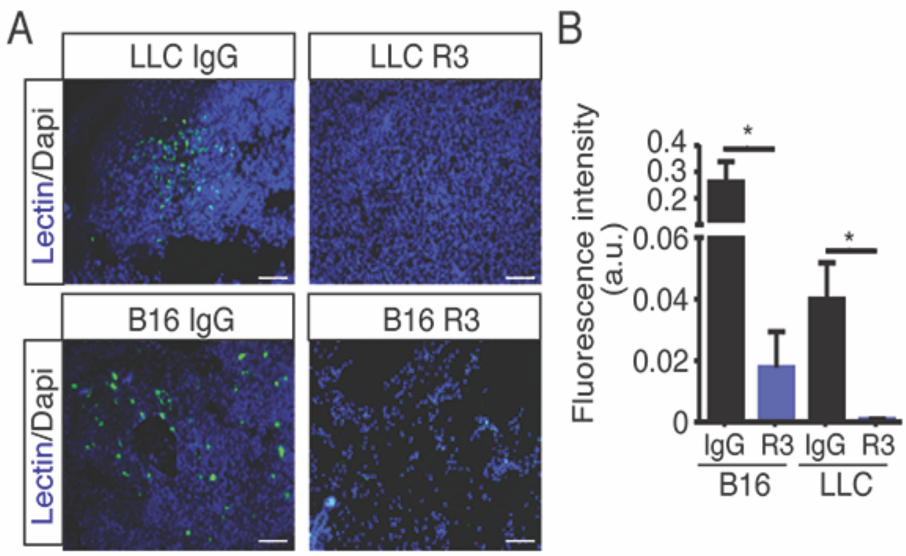

Supplementary Figure 3. Blocking lymphatic function abrogates tumour-AT lymphatic transport. A, Representative images of B16 and LLC tumor from mice treated with the anti-VEGFR-3 blocking antibody after intra-lymphatic injection of FITC-lectin (scale bar, 50µm). B, Quantification of lectin fluorescence intensity in B16 and LLC tumo3rs (*P<0.05).

Supplementary Figure 4

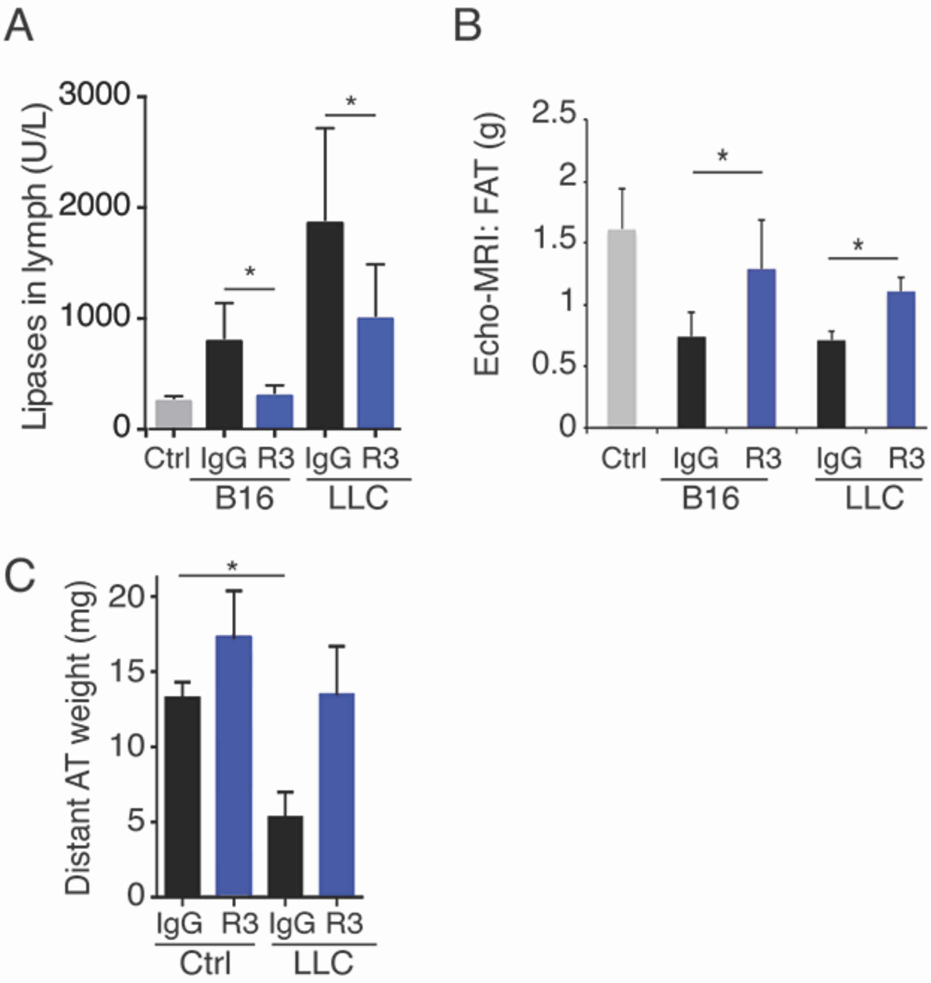

Supplementary Figure 4. Blocking VEGFR-3 reverse the AT loss.
A, Circulating pancreatic lipase dosage in lymph from B16- and LLC-tumor bearing mice treated with anti-VEGFR-3 or isotype control antibody (*p<0.05). B,Echo-MRI quantification of the fat mass in LLC and B16 tumour-bearing mice treated or not with the anti-VEGFR-3 (*p<0.05). C, The anti-VEGFR-3 inhibits tumour-distant (axillary) AT loss in LLC-bearing mice.

Supplementary Figure 5

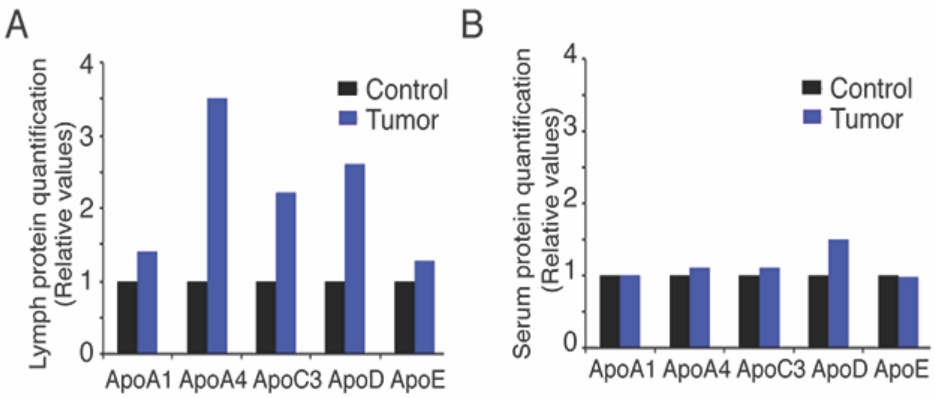

Supplementary Figure 5. Proteomic analysis of PKI mice lymph and serum.
A-B, Mass spectrometry measurement of apolipoproteins (Apo) A1, A4, C3, D, and E in the lymph (A) and in serum (B) from PKI mice compared to control littermates.

Supplementary Figure 6

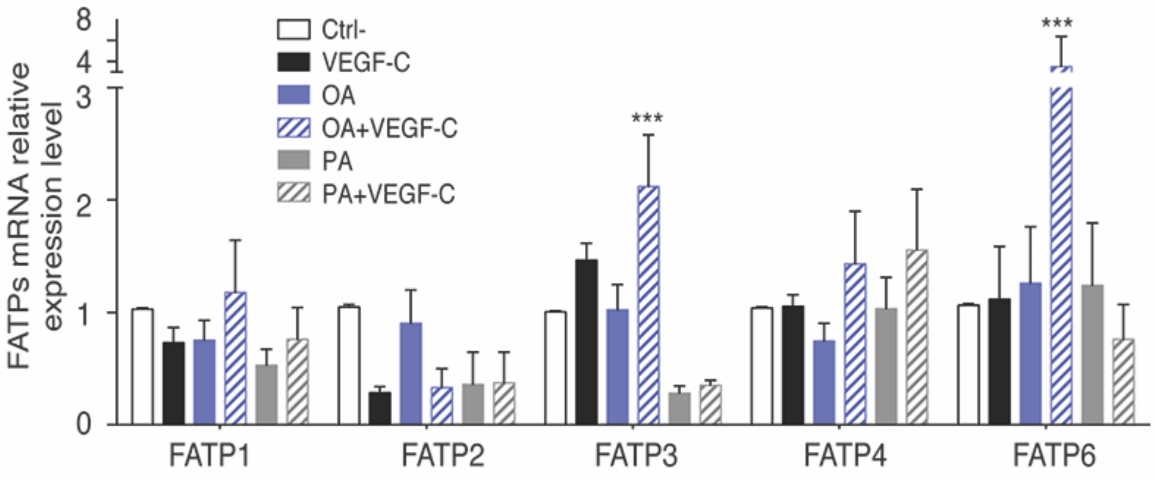

Supplementary Figure 6: FATPs expression in LECs.
FATP-1, -2, -3, -4 and 6 mRNA expression in OA or PA-stimulated LECs incubated with VEGF-C (***P<0.001 compared to Ctrl-).

Supplementary Figure7

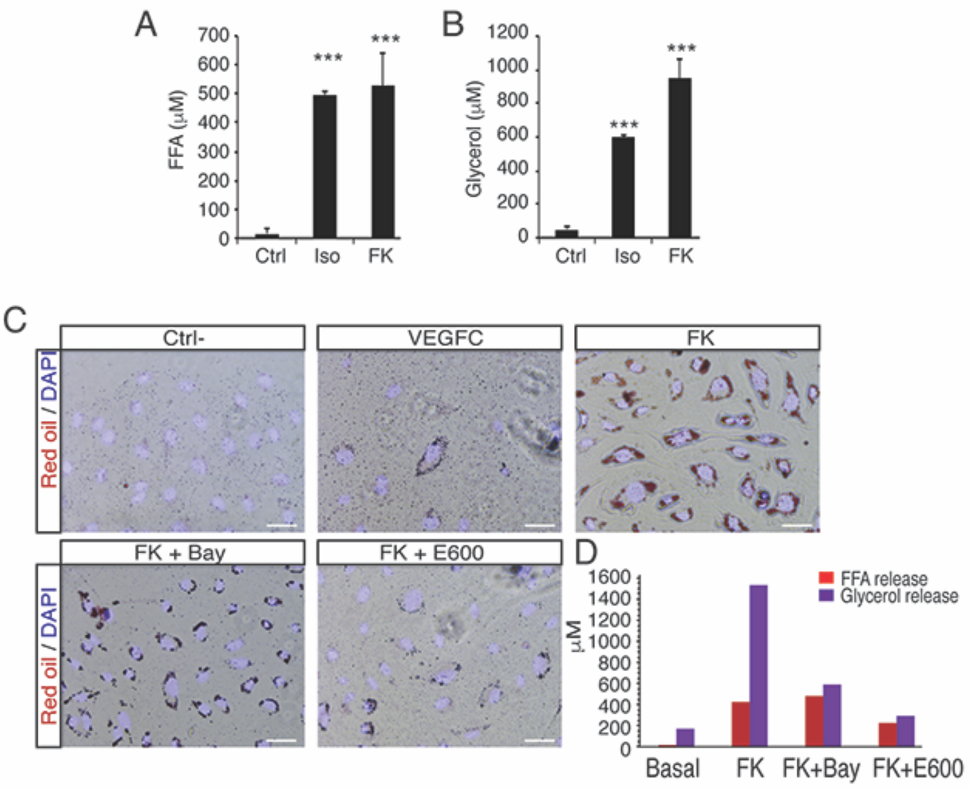

Supplementary Figure 7. FFA stimulate LEC function and FATP gene expression

A, Dosage of FFA released by adipocytes after isoproterenol (Iso) or forskolin (FK) stimulation. B, Dosage of Glycerol released by adipocytes after Iso or FK stimulation. C, Red oil uptake by LECs stimulated by FK-stimulated adipocytes conditioned media in presence of Bay and E600 lipases inhibitors. D, Dosage of FFA and Glycerol released by adipocytes after FK stimulation in presence of hormone sensitive lipase inhibitor (Bay) or non-selective lipase inhibitor (E600).
